# Supplementary material for: From Traditional Typing to Genomic Precision: Whole-Genome Sequencing of Listeria monocytogenes Isolated from Refrigerated Foods in Chile
Source: Foods. 2025 Jan 16;14(2):290. doi: 10.3390/foods14020290 (PMC11765429; doi:10.3390/foods14020290)
Supplement: Supplementary file 1 [file foods-14-00290-s001.zip › foods-3409888-supplementary.pdf]

Supplementary Materials

Table S1. CRISPR-Cas systems identified in *Listeria monocytogenes* genomes. The characteristic repeat sequences of the identified CRISPR arrays are shown, as well as their position in the genome.

Strains	SR Associated CRISPR-Cas System Type	Position	Number of Repeat Sequences	Number of Spacers	Repeat Sequence Consensus	*cas* Genes
511471	CAS-Type I-A	1202-1459	2	1	CATATTAGTAACGGAACTCGTATC	*cas3*
		2140-2215			GAAGTTTTATTGGTGATCTTCACA	*casRa*
511472	CAS-Type I-A	1202-1459	3	2	CATATTAGTAACGGAACTCGTATC	*cas3*
		10353-10428	2	1	TGAGAAGATCACCAATAAAACTTC	*casRa*
511475	CAS-Type I-A	107616-107873	3	2	CATATTAGTAACGGAACTCGTATC	*cas3*
510085	CAS-Type I-B	140268-140814	8	7	GATGTAAGTAATTTTGATACGAGTTCAGTAACTAATATG	*csa3, WYL*
510086	CAS-Type I-B	140268-140814	8	7	GATGTAAGTAATTTTGATACGAGTTCAGTAACTAATATG	*csa3, WYL*
		241328-241016	5	4	TTGTACTAGCCTTGAAGAAGTTGATGTAAGTAAT	
510087	CAS-Type I-B	140268-140814	8	7	GATGTAAGTAATTTTGATACGAGTTCAGTAACTAATATG	*csa3, WYL*
		241329-241017	5	4	TTGTACTAGCCTTGAAGAAGTTGATGTAAGTAAT	
510088	CAS-Type I-B	140268-140814	8	7	GATGTAAGTAATTTTGATACGAGTTCAGTAACTAATATG	*csa3, WYL*
510089	CAS-Type I-B	52588-53134	8	7	CATATTAGTTACTGAACTCGTATCAAAATTACTTACATC	*csa3*
		241329-241017	5	4	TTGTACTAGCCTTGAAGAAGTTGATGTAAGTAAT	
510090	CAS-Type I-B	481704-491259	7	6	ACTGAACTCGTATCAAAGGTACTTAAATCTAGCTCTTCAAGACT	*DinG, csa3,casR, WYL*
		127652-127880	4	3	AACATCTAAATTCGTTAGCTGATTGTTATCACAAGATAAA	
		276392-276938	8	7	CTTGAGGAGCTGGATGTAAGTAATTTTGATACGAGTTCAGTAACTAATATG	
510091	CAS-Type I-B	140268-140814	8	7	GATGTAAGTAATTTTGATACGAGTTCAGTAACTAATATG	*csa3, WYL*
510092	CAS-Type I-B	682317-682863	8	7	CATATTAGTTACTGAACTCGTATCAAAATTACTTACATC	*csa3, WYL*
		241328-241016	5	4	TTGTACTAGCCTTGAAGAAGTTGATGTAAGTAAT	
510225	CAS-Type I-B	682317-682863	8	7	CATATTAGTTACTGAACTCGTATCAAAATTACTTACATC	*csa3, WYL*
510228	CAS-Type I-B	140268-140814	8	7	GATGTAAGTAATTTTGATACGAGTTCAGTAACTAATATG	*csa3, WYL*
510231	CAS-Type I-B	241317-241005	5	4	TTGTACTAGCCTTGAAGAAGTTGATGTAAGTAAT	*csa3, WYL*
510237	CAS-Type I-B	241329-241017	5	4	TTGTACTAGCCTTGAAGAAGTTGATGTAAGTAAT	*csa3, WYL*
510250	CAS-Type I-B	140268-140814	8	7	GATGTAAGTAATTTTGATACGAGTTCAGTAACTAATATG	*csa3, WYL*
		241328-241016	5	4	TTGTACTAGCCTTGAAGAAGTTGATGTAAGTAAT	

Table S2. Spacer sequences that make up the CRISPR arrays, associated with sequences that correspond to bacteriophages.

Number of Spacer	Sequence (3′-5′)	Position	Phage Associated
**510085**			
1	GATGATACTCCCTATACAAAATGTCGACATCACGTGAACTCCTTGACCTACATTC	140307-140345	*Listeria phage LM 4-11-1* and PHAGE_Lister_A118
3	GATTATACATACCGTGCAAACAATTAACATGATCAGAACTTCTCGAACTACATTC	140463-140501	*Listeria phage LM 4-11-1* and PHAGE_Lister_A118
6	GATTATACATACGGTACAAACTTCTAACATGATCAGAACTTCTCGACCTACATTC	140697-140735	*Listeria phage LM 4-11-1* and PHAGE_Lister_A118
**510087**			
3	GATGATACGTCCGGTACAAACTTTTAACATGACCGGAACCTCTCGAACTACATTC	140463-140501	*Listeria phage LM 4-11-1 and PHAGE_Lister_A118*
6	GATGATACGTCCGGTACAAAATACCAACATGATCGGAACTTCTCGAACTACATTC	140697-140735	*Listeria phage LM 4-11-1 and PHAGE_Lister_A118*
**510088 and 510228-1**			
3	GATGATACGTCCGGTACAAACTTTTAACATGACCGGAACCTCTCGAACTACATTC	140463-140501	*Listeria phage LM 4-11-1 and PHAGE_Lister_A118*
6	GATGATACGTCCGGTACAAAATACCAACATGATCGGAACTTCTCGAACTACATTC	140697-140735	*Listeria phage LM 4-11-1 and PHAGE_Lister_A118*
**510089**			
1	GAATTTAGTTCGAAGAGGTCAAAAAGTAATAGTCTTTTGTACCGTCGGTATAATC	52627-52665	*Listeria phage LM 4-11-1 and PHAGE_Lister_A118*
3	GAATGTAGGTTGAAGAGTTCCCGCGATGTTAAAGATTTGTAAACTTGGTAAAGTC	52783-52821	*Listeria phage LM 4-11-1 and PHAGE_Lister_A118*
5	GAATGTAGGTCGAGAAGTTCTGATCATGTTAGAAGTTTGTACCGTATGTATAATC	52939-52977	*Listeria phage LM 4-11-1 and PHAGE_Lister_A118*
7	GAATGTAGGTCAAGGAGTTCACGGGATGTCGACAATTTGTATAGGGTGTATCATC	53095-53133	*Listeria phage LM 4-11-1 and PHAGE_Lister_A118*
**510091**			
3	GATGATACGTCCGGTACAAAATACCAACATGATCGGAACTTCTCGAACTACATTC	140463-140501	*Listeria phage LM 4-11-1 and PHAGE_Lister_A118*
6	GATGATACGTCCGGTACAAAATACCAACATGATCGGAACTTCTCGAACTACATTC	140697-140735	*Listeria phage LM 4-11-1 and PHAGE_Lister_A118*
